# Advances in Diagnostic Approaches for Viral Etiologies of Diarrhea: From the Lab to the Field

**DOI:** 10.3389/fmicb.2019.01957

**Published:** 2019-09-13

**Authors:** Yashpal Singh Malik, Atul Kumar Verma, Naveen Kumar, Nadia Touil, Kumaragurubaran Karthik, Ruchi Tiwari, Durlav Prasad Bora, Kuldeep Dhama, Souvik Ghosh, Maged Gomaa Hemida, Ahmed S. Abdel-Moneim, Krisztián Bányai, Anastasia N. Vlasova, Nobumichi Kobayashi, Raj Kumar Singh

**Affiliations:** ^1^Division of Biological Standardization, Indian Council of Agricultural Research-Indian Veterinary Research Institute, Izatnagar, India; ^2^ICAR-National Institute of High Security Animal Diseases, OIE Reference Laboratory for Avian Influenza, Bhopal, India; ^3^Laboratoire de Biosécurité et de Recherche, Hôpital Militaire d’Instruction Mohammed V, Rabat, Morocco; ^4^Central University Laboratory, Tamil Nadu Veterinary and Animal Sciences University, Chennai, India; ^5^Department of Veterinary Microbiology & Immunology, College of Veterinary Sciences, DUVASU, Mathura, India; ^6^Department of Microbiology, College of Veterinary Science, Assam Agricultural University, Guwahati, India; ^7^Division of Pathology, Indian Council of Agricultural Research-Indian Veterinary Research Institute, Izatnagar, India; ^8^Department of Biomedical Sciences, One Health Center for Zoonoses and Tropical Veterinary Medicine, Ross University School of Veterinary Medicine, Basseterre, Saint Kitts and Nevis; ^9^Department of Microbiology and Parasitology, College of Veterinary Medicine, King Faisal University, Al-Hufuf, Saudi Arabia; ^10^Department of Virology, Faculty of Veterinary Medicine, Kafrelsheikh University, Kafrelsheikh, Egypt; ^11^Department of Microbiology, College of Medicine, Taif University, Taif, Saudi Arabia; ^12^Department of Virology, Faculty of Veterinary Medicine, Beni Suef University, Beni Suef, Egypt; ^13^Institute for Veterinary Medical Research, Centre for Agricultural Research, Hungarian Academy of Sciences, Budapest, Hungary; ^14^Food Animal Health Research Program, Department of Veterinary Preventive Medicine, CFAES, Ohio Agricultural Research and Development Center, The Ohio State University, Wooster, OH, United States; ^15^School of Medicine, Sapporo Medical University, Sapporo, Japan

**Keywords:** enteric virus, infection, diagnosis, cell culture, molecular tests

## Abstract

The applications of correct diagnostic approaches play a decisive role in timely containment of infectious diseases spread and mitigation of public health risks. Nevertheless, there is a need to update the diagnostics regularly to capture the new, emergent, and highly divergent viruses. Acute gastroenteritis of viral origin has been identified as a significant cause of mortality across the globe, with the more serious consequences seen at the extremes of age groups (young and elderly) and immune-compromised individuals. Therefore, significant advancements and efforts have been put in the development of enteric virus diagnostics to meet the WHO ASSURED criteria as a benchmark over the years. The Enzyme-Linked Immunosorbent (ELISA) and Polymerase Chain Reaction (PCR) are the basic assays that provided the platform for development of several efficient diagnostics such as real-time RT-PCR, loop-mediated isothermal amplification (LAMP), polymerase spiral reaction (PSR), biosensors, microarrays and next generation sequencing. Herein, we describe and discuss the applications of these advanced technologies in context to enteric virus detection by delineating their features, advantages and limitations.

## Introduction

Advances in the enteric microbiology research have improved the understanding of etiology of infectious gastroenteritis, as well as the involvement and transmission modes of enteric pathogens. This has enabled the design of specific control strategies limiting the losses due to consequent severe infections. Although, bacteria and viruses are both responsible for gastroenteritis, the latter have had more impact on public health (Gunn et al., 2015). As of date, non-bacterial acute gastroenteritis, and respiratory infections are the leading causes of global deaths in both humans (mainly children) and animals (Dominguez et al., 2009; Bok and Green, 2012; Dhama et al., 2015). Since the identification of the first enteric virus, Norovirus (*Caliciviridae*), in 1972 using electron microscopy, a range of viruses, such as Rotavirus (*Reoviridae*), Picobirnavirus (*Picobirnaviridae*), Astrovirus (*Astroviridae*), enteric Adenovirus (*Adenoviridae*), Sapovirus (*Calciviridae*), Torovirus (*Coronaviridae*), Parechovirus, Bocavirus, and Aichivirus (*Picornaviridae*), and many more, have been found to be associated with gastroenteritis infections (Cheng et al., 2008; Dhama et al., 2009, 2014; Malik et al., 2011, 2014; Ahmed et al., 2014; Yip et al., 2014; Sidoti et al., 2015; Kattoor et al., 2017; Delmas et al., 2018; Kattoor et al., 2019). The enteric viruses known as of now along with their respective advanced diagnostic methods are tallied in Table 1. Although acute viral gastroenteritis is more common in immune-compromised and young individuals (Krones and Högenauer, 2012), it is also seen in the aged individuals, which may be due to changes in physiology and the waning of immunity with time (Estes and Kapikian, 2007).

**TABLE 1:** A list of diverse enteric viruses along with respective advanced diagnostic methods.

**Enteric viruses**	**Methodologies**	**References**
Sapelovirus	Sanger sequencing, RT-qPCR, RT-LAMP	Abe et al., 2011; Chen et al., 2014; Wang et al., 2014
Parvovirus	Metagenomics, lateral flow strip-recombinase polymerase amplification (LFS-RPA), multiplex TaqMan real-time PCR	Phan et al., 2012; Liu et al., 2018; Sun et al., 2018
Astrovirus	RT-PCR, sanger sequencing, metagenomics	Chu et al., 2012; Phan et al., 2014; Guan et al., 2018
Rotavirus	Hybridization (DIG) probe, surface enhanced raman spectroscopy, RT-LAMP, RT-PCR, novel enzyme immunoassay, paper-LAMP	Minakshi, Prasad et al., 2005; Basera et al., 2010; Driskell et al., 2010; Xie et al., 2012; Kumar et al., 2016; Ye et al., 2018
Coronavirus	Pyrosequencing, RT-LAMP	Honkavuori et al., 2014; Wang et al., 2018
Bufavirus	Metagenomics, sanger sequencing	Smits et al., 2014; Kemenesi et al., 2015
Sakobuvirus and feline bocavirus 2	Metagenomics	Ng et al., 2014
Sapoviruses	Metagenomics, digital RT-PCR	Sachsenröder et al., 2014; Varela et al., 2018
Norovirus	Metagenomics, electrochemical biosensor, aptamer based *in situ* capture RT-qPCR	Nasheri et al., 2017; Baek et al., 2019; Liu D. et al., 2019
Calhevirus 1	Metagenomics	Sasaki et al., 2015
Bocavirus	RT-PCR and sanger sequencing	Gunn et al., 2015
Canine vesivirus	RT-PCR and sanger sequencing	Martella et al., 2015
Torovirus	RT-PCR and sanger sequencing, RT-LAMP	Zhou et al., 2014; Liu et al., 2016
Picobirnavirus	RT-PCR and sanger sequencing	Bányai et al., 2014; Malik et al., 2017
Adenovirus	RT-PCR and sanger sequencing	Pabbaraju et al., 2010
Kobuvirus	TaqMan RT-qPCR	Zhu et al., 2016
Smacoviruses	Metagenomics	Ng et al., 2015
Mammalian orthoreoviruses 3	RT-PCR and sanger sequencing	Lelli et al., 2016
Cosavirus	RT-qPCR	Okitsu et al., 2014
Salivirus	RT-PCR and sanger sequencing	Ayouni et al., 2016
Passerivirus	RT-qPCR	Pankovics et al., 2018
Posavirus (porcine stool-associated RNA virus)	RT-PCR and sanger sequencing	Chen et al., 2018

New viruses are emerging at a faster pace, apparently as a feature of their rapidly changing genetic makeup due to the accumulation of point mutations, reassortments or recombinations (Malik et al., 2016; Kattoor et al., 2017). For an example, a new porcine coronavirus, has emerged through recombination between the transmissible gastroenteritis virus and a porcine epidemic diarrhea virus (Boniotti et al., 2016). Enteric viral diseases are diagnosed by identifying the causative viral agents in feces/body fluids or viral antigens and/or antibodies in the serum of patients. Conventional methods to achieve this, however, are either inefficient, cumbersome or time consuming, because of the pace of change of the virome. There were not much significant approaches available in the past, and in the recent time various techniques have come up offering a modern field for advances in bio-techniques for the easy, quick and reliable diagnosis and discovery of new viruses. In clinical laboratories, polymerase chain reaction (PCR)-based assays are considered as gold-standard for the detection of viruses, but when it comes to multiple detections of similar types of viruses simultaneously, variations in the properties of viral nucleic acids make the amplification difficult (Fout et al., 2003; Fong and Lipp, 2005). Among different techniques used to explore new viruses, such as conventional and next-generation sequencing, metagenomics has been a promising approach to study the unrevealed viral genomes since more than a decade (Garza and Dutilh, 2015; Martinez-Hernandez et al., 2017). This allows researchers to study the genetic material directly from pooled samples and bypass the need for culturing the virus *in vitro* as well. Virome capture sequencing is another approach for vertebrate viruses, in which several million probes covering the genomes of several viral taxonomies are used to enrich virus targets (Briese et al., 2015). A new metagenomic sequencing method, ViroCap, based on the target nucleic acid capture and enrichment detects viral sequences with up to 58% variation from the references used to select capture probes (Wylie et al., 2015).

**FIGURE 1:**
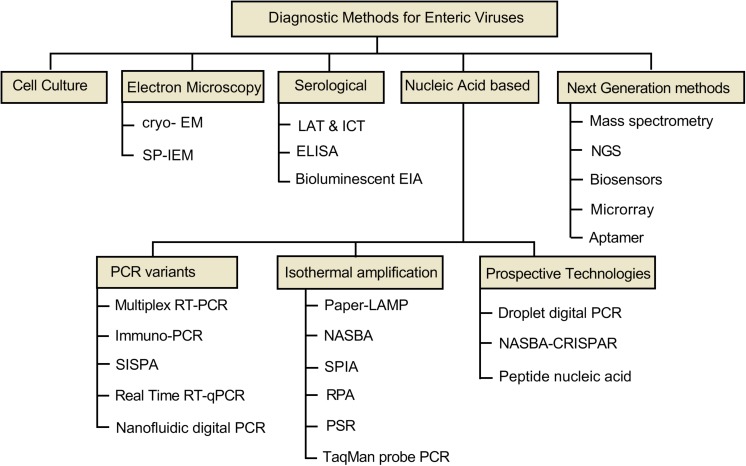
A schematic representation of diagnostic methods for enteric viruses. The diagnostic assays are classified in five major categorizes (i) cell culture, (ii) electron microscopy, (iii) serological methods, (iv) nucleic acid methods, which further have three sub-classifications, and (v) next-generation methods. The techniques are individually dealt under different sections.

Nevertheless, several diagnostic methods have been developed over the last two decades, seeing the constant evolution of viruses, newer, sensitive, efficient, and rapid diagnostics are still warranted for the effective diagnosis (Liu et al., 2007; Saminathan et al., 2016). This paper systematically describes and discusses the features, advantages and limitations primarily of advanced diagnostic tools devised for the sensitive and quick detection of enteric viruses worldwide (Figure 1).

## Enteric Virus Detection Methods

### Cell Culture System for Enteric Virus Isolation/Propagation

Isolation of the enteric viruses in cell culture system from fecal samples is the most conventional way of confirmatory diagnosis. Although the cultivation of viruses in cell culture is time and labor intensive, taking from days to weeks before the virus is adapted to cell culture; it is still the ideal and gold standard method for the virus detection worldwide. Many new cell lines have been developed for easy propagation of enteric viruses and are given in Table 2.

**TABLE 2:** Cell cultures/cell lines in-use for the isolation and propagation of enteric viruses.

**Virus**	**Cell line/culture**	**Origin of cell line**	**References**
Rotavirus	MA104	African green monkey epithelial cell	Otto et al., 2015
	HT-29	Human colon carcinoma cell line	Holloway et al., 2018
	Colonic Caco-2	Colon epithelial cells of Human	Bautista et al., 2015
Sapovirus	LLC-PK1	Primary porcine kidney cells or a porcine kidney cell line	Oka et al., 2015
Sapelovirus	PK-15	Porcine kidney cells	Zhao et al., 2019
Reovirus	Vero cell line	African green monkey	Islam et al., 2003
Astrovirus	HEK	Human embryo kidney	Méndez et al., 2014
	Colonic Caco-2	Colon epithelial cells of Human	Park et al., 2012
Coronavirus	Continuous cell line	Human rectal adenocarcinoma	Dea et al., 1989
Bocavirus	HEK293	Human embryonic kidney	Huang et al., 2012
Adenovirus	A549	Human lung carcinoma	Hematian et al., 2016
Norovirus	BJAB cell line	Human B cell lines	Jones et al., 2015
	iPSC–derived IECs	Human induced pluripotent stem cell	Sato et al., 2019

New methods for the easy cultivation, better preservation and isolation of viruses have been developed; for example, cryopreserved cell culture, virus isolation in co-cultured cells and virus identification in transgenic cell lines. Inclusion of genetic elements in the transgenic cell lines has helped in the rapid and accurate detection of some viruses. For culturing gastrointestinal viruses in the laboratory, induced intestine-like human intestinal organoids (iHIOs) with differentiation of human embryonic or induced pluripotent stem cell lines have been used successfully for culturing rotavirus (McCracken et al., 2011; Spence et al., 2011). This also increases the potential for successful isolation and propagation of other enteric viruses, although there is still a need to develop better isolation or cultivation methods for enteric viruses. For other enteric viruses viz. Calicivirus, except for murine Norovirus and porcine Sapoviruses (Cowden strain), establishment of an efficient human Norovirus and Sapovirus cultivation system is still lacking (Greenberg et al., 1982; Sidoti et al., 2015). Jones et al. (2015) were the first to report the *in vitro* cultivation of GII.4-Sydney human Norovirus strain in B cell line (BJAB cell line) and achieved the modest level of viral output, ranging from 0.5 to 3.5 logs. Four days were found optimum for infection and analysis assays. Recent attempts to grow human Noroviruses have been established in human induced pluripotent stem cells derived intestinal epithelial cells (iPSC–derived IECs) (Sato et al., 2019).

### Electron Microscopy

Pioneering work for pathogen identification was started with the visualization of the virus under an electron microscope (EM). Electron microscope came into existence with the efforts of Knoll and Ruska (1932), and Tobacco Mosaic Virus was the first virus to be visualized. Before 1970, >80% of gastroenteritis cases could not be etiologically diagnosed. These cases were attributed to either weaning, or most often, idiopathic causes. In 1971, the first enteric virus was observed under EM. Thereafter, from 1972 onward, microbiologists began to examine fecal samples from patients with acute gastroenteritis using EM, and within a decade, a collection of novel enteric viruses had been discovered (Leland and Ginocchio, 2007). With the use of EM, Sapoviruses were discovered for the first time in 1976, from human cases of diarrhea, and later on from different species including pigs, mink, dogs, sea lions and bats. Due to the characteristic “Star of David” appearance of the surface morphology of Sapoviruses under EM, they are easily differentiated from other viral pathogens causing gastroenteritis e.g., Rotavirus, Parvovirus, Astrovirus, and Adenovirus (Oka et al., 2015). The EM continues to be an important tool in the diagnosis of enteric virus’s infection and is frequently used to resolve discrepancies in results from other techniques, although it is only practical when a few samples are to be examined. Moreover, it suffers from low sensitivity, while also needing costly equipment and trained personnel. EM is useful in detecting a variety of enteric viruses quickly on the same day of sample submission, but the virus particle count should not be less than 10^6^/ml per gram fecal specimen (Beniac et al., 2014).

Immunoelectron microscopy (IEM) technique is based on antigen-antibody reaction, which increases sensitivity 100-fold when a specific antibody connected to an electron dense marker detects antgen-antibody complex. This technique has been used for the detection of Rotavirus and can even differentiate between morphologically indistinguishable A, B, and C Rotaviruses (Morinet et al., 1984).

Solid-phase IEM (SPIEM) is a modification of IEM in which the viral particles are captured directly over the solid surface of a grid. The SPIEM method has been suggested for diagnosis of Norovirus using protein A, goat anti-human immunoglobulin M (IgM), and human serum (Lewis et al., 1988). At least 10^6^ virus particles per gram fecal suspension are required for visualization, and × 50,000–60,000 magnification is normally used. The major drawbacks of SPIEM include the low sensitivity and requirement for expensive instrumentation, as well as the cost of its maintenance.

Hepatitis A virus, the Norovirus, and the Rotavirus particles could easily be differentiated by rapid negative-contrast diagnostic EM and could be recognized in feces or by their reaction to a specific antisera (Gentile and Gelderblom, 2014). Although, the direct method is as sensitive and accurate as cell culture, it requires a long time because each fecal sample must be prepared individually and therefore, not adapted to urgent testing when gastroenteritis outbreaks occur. The use of convalescent sera in IEM for the detection of new viral agents is also noted (Lavazza et al., 2015). This approach is particularly useful where no indications are available and other diagnostic methods fail. Important porcine enteric viruses (Rotavirus, Torovirus, and Circovirus), Astrovirus in turkeys, and Parvovirus in pheasants were targeted for successful detection using this methodology.

Recently, a new and rapid method for the detection of Rotavirus has been developed where monoclonal antibodies functionalized with magnetic microparticles were used to capture, concentrate, separate and detect infectious Rotavirus particles in distilled and drinking water samples (Villamizar-Gallardo et al., 2017). Further, confocal microscopy helps in the identification of Rotavirus, taking advantage of fluorescent microparticles. Functionalizing fluoromagnetic microparticles with anti-rotavirus monoclonal antibodies would form a fast, simple, sensitive, rapid, and reliable technique for the detection of other enteric viruses too (Villamizar-Gallardo et al., 2017).

### Serological Methods

Although, over the years the serological methods have turned out to be very productive, the complexity of gastrointestinal virome represents a challenge for the use of such methods. To overcome this, new advances in pathogen detection, including the enteric viruses have shifted toward exploring the immune components, naming serological methods to speed-up the viral diagnosis. The latex agglutination test (LAT) is among the more favored, easiest and rapid methods, and thus is used nowadays as a pen-side test for the identification of several enteric pathogens. Latex beads pre-coated with antibodies against a specific virus are agglutinated when they come into contact with the respective viruses present in the tested sample, forming large agglutinates. This technique was found to be more specific, more sensitive, faster and cheaper in detecting Rotavirus during acute illness when compared to EM or ELISA (Ferreira et al., 2006). Avian IgY antibodies, which have several advantages over the mammalian IgG, have also been employed in immune-chromatography test (ICT) for the rapid detection of Rotavirus (Reschova et al., 2000). ICT-based virus detection strips/kits are also available in the market for the identification and direct detection of Norovirus, Rotavirus, and Astrovirus in fecal samples with good sensitivity and specificity ranging between 90–95% (Khamrin et al., 2009).

One of the most widely used and simple serological diagnostic methods is ELISA and its several modifications are in-use in the form of commercial kits for enteric viruses. Thachil et al. (2015) developed a modified IgG ELISA, based on the S1 portion of the spike protein of porcine delta Coronavirus with high sensitivity (91%) and specificity (95%). An ultrasensitive and fully automated bioluminescent enzyme immunoassay (BLEIA) has been developed for the detection of Norovirus capsid antigen, labeled with firefly luciferase, which possesses a good yield of quantum energy providing high sensitivity (Sakamaki et al., 2012). This method is very useful in the diagnosis of asymptomatic gastroenteritis and has been successful even in the detection of various genotypes of Norovirus (Shigemoto et al., 2014). The results are comparable to other sensitive methods such as ELISA and immunochromatography, can also be applied in the rapid diagnosis of other enteric viruses (Suzuki et al., 2015).

The author’s lab has developed a novel enzyme immunoassay for the detection of Rotavirus A (RVA) antigen in fecal samples of multiple host species with high diagnostic sensitivity and specificity. The concept of this assay was based on the detection of conserved VP6 protein using anti-recombinant VP6 antibodies as capture antibodies and anti-multiple antigenic peptide (constructed from highly immunodominant epitopes within VP6 protein) antibodies as detector antibodies. This assay has already been validated on the fecal samples of four hosts (bovine, porcine, poultry, and human) and also showed a high concordance with diagnostic RT-PCR (Kumar et al., 2016). Recently, a sensitive sandwich ELISA for the detection of NoV genogroup II has been developed that provided an improved detection limit of 13.2 copies/mL fecal suspension for Norovirus as compared to gold-immunoassay (1000-fold) and horseradish peroxidase-based ELISA (100-fold). This assay utilized anti-NoV genogroup II antibodies as capture antibodies and antibodies conjugated to silver ion-incorporated gold nanoparticles (as detection antibody) (Khoris et al., 2019). This ELISA format delivered a significant improvement of sensitivity compared to commercial immunoassay kits.

### Nucleic Acid Based Methods

Nucleic acid purification directly from the fecal samples is a first and key step for rapid molecular diagnosis of enteric viruses. The ready-to-use kits are now widely commercialized that primarily use chemical extraction followed by spin column binding and purification, ensuring detection of low-level enteric viruses in fecal samples (Kowada et al., 2018). Additionally, some kits use internal controls to measure the efficiency of critical steps for viral genome quantification. To validate the behavior of viral genome during the nucleic acid extraction procedures, the use of a non-pathogenic virus with structural characteristics similar to those of the target virus is a prerequisite (Costafreda et al., 2006). The nucleic acid extraction from the samples such as wastewater, fecal samples and bio-solids become tricky as they are high in PCR inhibitors. However, the inclusion of inhibitors removal technology in the nucleic acid extraction protocol has solved this problem (Iker et al., 2013). This scientific progress is fundamental for an efficient molecular diagnostic for enteric viruses.

Owing to high specificity and sensitivity of molecular methods, most laboratories employ these methods for accurate diagnosis of viruses (Steyer et al., 2016). These methods largely involve the amplification of DNA or RNA by various nucleic acid amplification techniques. Till now, many nucleic acid amplification techniques have been devised for rapid and accurate diagnosis of enteric viruses and certain unique advanced techniques have been listed in Table 3.

**TABLE 3:** Various diagnostic techniques and their recent modifications.

**S. No.**	**Techniques**	**Principle**	**Modifications**	**References**
1	NASBA	Useful for RNA detection, utilizes the activity of reverse transcriptase, T7 RNA polymerase and RNase H. Two primers are used, one for initial binding of T7 RNA polymerase and second primer binds to the cDNA formed.	Paper-based cell-free systems and synbody-based viral enrichment	Ma D. et al., 2018
2	Real-time RT-qPCR	Real-time amplification of DNA/RNA using fluorescent reporter	Nanofluidic RT-qPCR, Multiplex RT-qPCR, Aptamer based RT-qPCR	Monteiro and Santos, 2017; Wongboot et al., 2018; Liu D. et al., 2019
3	LAMP	Isothermal amplification of a targeted sequence in loop mediated displacement	Real-time RT-LAMP, Paper-LAMP	Wang et al., 2018; Ye et al., 2018
4	PLA	Amplification of ligated oligonucleotides by connector sequence, bound to antibodies	PEA (proximity extension assay)	Lundberg et al., 2011; Assarsson et al., 2014
5	PCR	Amplification of sequence in presence of Taq polymerase containing stages of annealing and extension per cycle	Multiplex PCR	Liu G. et al., 2019
6.	RPA	Isothermal amplification using a recombinase, a single-stranded DNA-binding protein (SSB) and strand-displacing polymerase	Lateral flow strip-RPA	Liu et al., 2018

#### Polymerase Chain Reaction (PCR)

Many PCR variants/modifications have evolved from the conventional PCR to meet the demand of highly sensitive and specific diagnostic assays for enteric viruses. For example, integration of cell culture with reverse transcription (RT)-PCR allowed the detection of viruses that are either slow growing or fail to produce a cytopathic effect (Rigotto et al., 2010; Ryu et al., 2015). To differentiate the infectious enteric viruses from non-infectious ones, Parshionikar et al. (2010) used propidium monoazide (PMA) dye in conjunction with RT-PCR. This method is based on the penetration of the PMA dye through damaged capsids of non-infectious viruses and then it’s covalently binding to viral RNA on exposure to visible light. This covalent binding makes RNA unavailable for amplification by RT-PCR.

**FIGURE 2:**
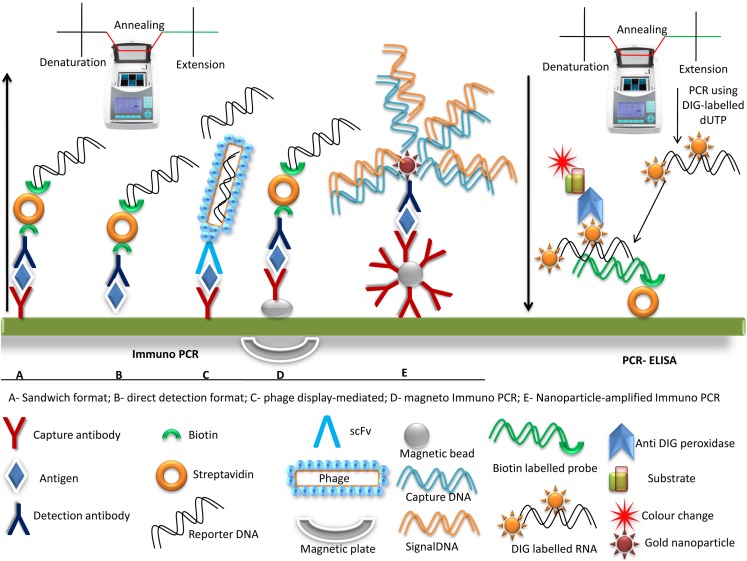
A schematic representation of Immuno PCR and PCR-ELISA. There are different immuno PCR platforms available as shown in figure **(A–E)**. The Immuno PCR format starts with an immune assay followed by PCR. Immuno PCR is similar to ELISA except terminal DNA is amplified by a PCR. **(A)** Sandwich format of Immuno PCR, **(B)** Direct format of antigen detection, **(C)** Phage mediated where single chain variable fragments (scFv) and DNA are carried by the phage and upon heating DNA is released from the phage, **(D)** Magneto Immuno PCR, where the antibody is captured on a magnetic bead with the rest of the protocol being similar to the sandwich format, **(E)** Nanoparticle amplified immune PCR where magnetic beads capture several antibodies which uses gold nanoparticle in a sandwich pattern to amplify DNA by PCR. PCR-ELISA starts with PCR followed by an immune assay and detecting the color production. The target gene is amplified in the presence of DIG-dUTP. Employing the avidin biotin affinity, the amplified gene is immobilized on plate and finally using substrate, enzyme and conjugate, color production is visualized.

Significant improvements through the modifications in RT-PCR have been made over the years for the detection of difficult to culture enteric viruses or multiple enteric viruses. One of such modification is immune-PCR that increases the sensitivity by 200 times, minimizes the effect of inhibitory components, reduces labor, and is very helpful in detecting viruses in stool samples where viruses are present in very low amounts (Figure 2). A major advantage in its application is that several viral nucleic acids can be detected simultaneously. The turn-around time is very short but the high background noise represents a major hindrance in its application. This is all because of the non-specific binding of chimeric DNA on the walls of wells. Bonot et al. (2014) applied this principle in the detection of human Adenovirus. In a combination of PCR and ELISA, DIG-labeled target amplicons are hybridized to biotinylated probes incubated with streptavidin on a microtiter plate. Enzyme-linked anti-DIG antibodies allow for the colorimetric quantification of the PCR product after adding the substrate (Figure 2). Its application has been utilized for detection Aichi virus (Yamashita et al., 2000) and Norovirus (Schwab et al., 2001).

For detection of highly divergent viruses or new viruses of unknown sequence, sequence independent single primer amplification (SISPA) could be useful. In this technique, linker/adaptors of the known sequence are ligated at both the ends of unknown sequence, and amplification is done by designing the primers against these linker/adapters. The SISPA technique has already been used successfully for detection of an unknown genomic sequence of Norovirus associated with gastroenteritis (Reyes and Kim, 1991), human Rotavirus C genome (Lambden et al., 1992; Lambden and Clarke, 1995), non-cultivable Rotavirus (Lambden et al., 1992), and sequencing of Picobirnavirus (Karlsson et al., 2016). A multiplex RT-PCR assay for simultaneous detection of 14 diarrheal viruses commonly associated with acute gastroenteritis in humans was developed (Thongprachum et al., 2017). The sensitivity of this assay was comparable to monoplex RT-PCR assay. Similarly, for the accurate diagnosis and differentiation of porcine enteric viruses such as Porcine Epidemic Diarrhea Virus (PEDV), Transmissible Gastroenteritis Virus (TGEV), Porcine Group A Rotaviruses (RVA), Porcine Group C Rotaviruses (RVC), and Porcine Circovirus 2 (PCV2), a multiplex PCR was developed. The detection limits of the assay were 5 copies for PEDV, TGEV, RVC and PCV2 and 50 copies for RVA for the singleplex assays while 50 copies when all five viruses were multiplexed (Liu G. et al., 2019).

#### Real-Time RT-PCR (RT-qPCR)

Real-time RT-PCR is an excellent tool for the rapid sensitive detection and quantification of viruses, where the amplified product produced during each cycle is quantified either by using SYBR Green or by various fluorescent probe chemistries. A Luminex xMap technology offers a high throughput platform for development of multiplex RT-qPCR assay and is a bead-based multiplex system based on flow cytometry. The multiplex luminex assays haven been successfully developed and evaluated for simultaneous detection of many enteric viruses (Liu et al., 2012; Hamza et al., 2014; Jiang et al., 2014). These assay showed a better sensitivity than conventional RT-PCR and comparable to RT-qPCR. The detection of different antigenic types of viruses, and multiple viruses in a single assay is always preferred to solve the complex etiology posed by diverse enteric viruses. A RT-qPCR assay for the detection of antigenic types of RVA (Anaya-Molina et al., 2018), and CPV (Sun et al., 2018) with high accuracy has been reported. Besides, a multiplex RT-qPCR assay was developed for the detection of different enteric viruses, namely Astrovirus, Adenovirus, Rotavirus A, C, Sapovirus, and Enterovirus from stool samples. However, this assay could not detect Norovirus (Kowada et al., 2018). Another RT-qPCR assay to simultaneously detect diverse 19 enteric pathogens with high sensitivity has also been reported recently (Wongboot et al., 2018).

The major drawback in these assays is inter-laboratory variability due to standard curve creation and use of different standard materials. To overcome these limitations, digital PCR (dPCR) could be used as an alternative methodology to RT-qPCR. The dPCR works by segregating a sample into thousand reactions followed by amplification of targets and their calculation. In this way, it also reduces the level of inhibitors commonly associated with complex fecal samples. Using the same principle, a nanofluidic RT-qPCR was investigated for the identification of 19 human enteric viruses and it showed a better sensitivity compared to RT-qPCR (Coudray-Meunier et al., 2016; Monteiro and Santos, 2017).

##### Real-time polymerase chain reaction based on primer probe energy transfer

This method is an example of combined detection and quantitation of viral genome copies. It employs the principle of primer-probe energy transfer (PriProET) and can simultaneously detect viruses with large variations, with a sensitivity that allows the detection of as few as ten genome copies of Feline Coronavirus (FCoV) (Hornyák et al., 2012). The quantitative sg-mRNA detection method revealed a more than 10,000–50,000 times increase in the M gene sg-mRNA in organ materials of feline infectious peritonitis cases, compared to those of the enteric FCoV variants present in the feces of normal healthy cats (Hornyák et al., 2012).

#### Isothermal Nucleic Acid Amplification Based Assays

##### Nucleic acid sequence-based amplification (NASBA)

Nucleic acid sequence-based amplification is a two-step process in which the first step is denaturation and second is temperature labile polymerase dependent isothermal amplification (Compton, 1991). For real-time observation, fluorochrome labeled probes is added to the reaction. Mo et al. (2015a) developed a multiplex real-time nucleic acid sequence-based amplification (RT-NASBA) system for the concurrent detection of Rotavirus A and Norovirus genogroup II/Astrovirus. The limits of detection were 7, 100, and 200 copies per reaction for Rotavirus A, Norovirus genogroup II and Astrovirus, respectively. This multiplex RT-NASBA was 10 to 100 times more sensitive than conventional multiplex RT-PCR and, gives an advantage for the detection of multiple infections. RT-NASBA needs much less time than RT-PCR because the former is conducted under isothermal conditions where no time is consumed for heating and cooling, no separate RT steps are needed, and copies are produced faster than RT-PCR, and this makes RT-NASBA a more sensitive and specific method. Moreover, Dunams et al. (2012) developed an *in situ* nuclease-resistant molecular beacon-based assay for simultaneous detection of human Adenovirus and Echovirus. RT-NASBA has been proved to be more efficient than the conventional RT-PCR and TaqMan RT-PCR assays (Mo et al., 2015b).

##### Loop-mediated isothermal amplification (LAMP)

**FIGURE 3:**
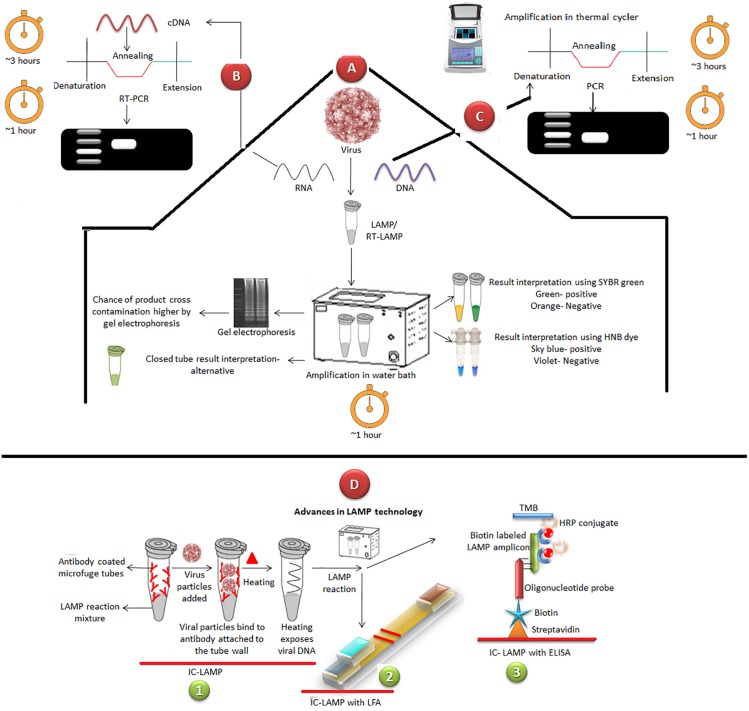
Comparison between thermal cycling based PCR and isothermal LAMP assays employed for the detection of viruses and a glimpse on recent advances in LAMP assays. **(A)** LAMP does not require sophisticated instruments; a water bath is sufficient and time for completion is approximately 1 h. Results can directly be visualized by addition of SYBR Green or hydroxynapthol blue (HNB) or calcein dyes. Gel electrophoresis can also be employed where a ladder-like pattern can be visualized. Additionally, closed tube LAMP assays are being developed. **(B)** RT-PCR starts with RNA extraction from virus and cDNA preparation followed by repeated cycling using a sophisticated thermal cycler and procedure takes approximately 3 h to complete. Subsequently, to analyze the results, gel electrophoresis has to be carried out, which takes additional 1 h. **(C)** PCR starts with DNA extraction from virus followed by all cycles stated in B step and takes 3–4 h to complete the procedure. **(D)** Advances in LAMP, like Immunocapture LAMP (1) assays are being developed where antibodies against the virus are coated in the tubes and the LAMP mixture is in lyophilized form at the bottom of the tubes. On virus sample addition it bind to the specific antibodies and, upon heating, their DNA is exposed which then mixes with the LAMP mixture. Results can be visualized using LFA (2) or ELISA (3) platforms.

Loop-mediated isothermal amplification is an accurate, rapid and inexpensive diagnostic method that selectively amplifies the target nucleic acid under isothermal conditions, generally around 60°C. This method is based on the use of a set of four to six specially designed primers. In LAMP, initial template denaturation is not required, and the development of strand-displacement polymerases has decreased the reaction time to less than 30 min. The whole reaction occurs in a single tube. Hydroxynaphthol blue (HNB) can be used in the colorimetric analysis of LAMP reactions since it can be mixed prior to the amplification of the nucleic acid obviating the need to open the assay samples to add the dye, and thus helping to reduce cross-contamination (Figure 3). Other methods to prevent product cross-contamination include agar dye capsule, paraffin or tin foil (Karthik et al., 2014). Besides, a method that could reduce the LAMP reaction time to half has also been described that utilized an additional set of primers termed as loop primers (Nagamine et al., 2002). The combination of this rapid method of LAMP utilizing loop primers and detection of final products using HNB would be useful for rapid on-field genetic detection of enteric viruses.

Reverse transcription-LAMP assay was developed for the detection of Noroviruses and the sensitivity was shown to be comparable with commercial kits (NVGI and NVGII detection kits; Eiken Chemical Co., Ltd., Japan) (Yoda et al., 2007). RT-LAMP was used to detect bovine Rotavirus using Rotavirus VP6 gene as a target for primers. The probe design was specific and no cross reactivity was observed with other bovine pathogens. The sensitivity of the test was 3.32 copies of bovine Rotavirus (Xie et al., 2012). In a similar study, but using a different strategy, RT-LAMP was exploited for the rapid detection of human Rotavirus targeting the NSP4 gene. The sensitivity of the RT-LAMP assay was 1.26 × 10^4^ copy numbers (Malik et al., 2013). Real-time RT-LAMP was also developed for the diagnosis of porcine epidemic diarrhea and a novel Swine Acute Diarrhoea Syndrome-Coronavirus (SADS-CoV) while maintaining equivalent sensitivity with RT-qPCR (Yu et al., 2015; Wang et al., 2018).

To increase the sensitivity and simplicity of existing RT-LMAP, various modifications or combinations with other techniques have been devised. One of such modification is Immunocapture (IC)-LAMP, where canine Parvovirus was first captured by surface antigen specific antibody followed by isothermal amplification of its genome or IC-LAMP with ELISA where biotin-labeled probes were designed for hybridization with LAMP amplicons on streptavidin-coated wells for visual confirmation of canine parvovirus (Figure 3; Sun et al., 2014, 2017). Another modification is LAMP- lateral flow dipstick (LAMP-LFD) which utilizes FITC labeled probes for rapid and visual detection of canine Parvovirus and human Enterovirus, Coxsackie virus (Sun et al., 2014; Yan et al., 2015). The sensitivity and simplicity of these methods were much superior as compared to conventional PCR.

Recently, a simple paper-based test for the point-of-care diagnosis of Rotavirus A that utilizes the isothermal amplification technique (Paper-LAMP) has been developed. This paper-based test could facilitate nucleic acid extraction within 5 min, with a further 25 min to amplify the target sequence, and the result can be visible to the naked eye immediately afterward or quantitative by the UV–Vis absorbance. The detection limit for Rotavirus A was found to be 1 × 10^3^ copies/mL. This low-cost method does not require extra equipment and is easy to use either in a lab or at the point-of-care (Ye et al., 2018).

##### Single primer isothermal amplification (SPIA)

This isothermal amplification technique utilizes the chimeric DNA/RNA sequence, RNase H, to degrade the 3′ end of the RNA to serve as a primer, and blockers to terminate the reaction. The blocker is a short oligonucleotide sequence, downstream of sequence amplification, to stop the further extension. On amplification, the RNA sequence gets digested by RNase H and the vacated site is occupied by primers in the next round. SYBR Green II is used in the identification as it binds to single-stranded DNA (Wang et al., 2016). This method is sensitive enough to amplify just 1 ng of mRNA. Its main drawback however is that it needs to be confirmed on agarose gel electrophoresis since RNase H makes the reaction unamplifiable. This technique is magnificent in the detection where a robust primer design has been not possible. Hu et al. (2015) applied this technique in combination with microarray for detection and genotyping of human Norovirus.

##### Recombinase polymerase amplification (RPA)

Recombinase polymerase amplification employs a bacterial recombinase and binding proteins and is generally carried out at slightly higher than normal temperature, since a low temperature may give non-specific amplification artifacts. It has brought about a breakthrough in the detection of nucleic acids and is an excellent substitute to PCR. The process takes just 30 min and does not need any thermal denaturation of the template. A high fidelity, portability, cost efficiency, simplicity, sensitivity, and tolerance to inhibitors, make this an important technique, and it is easy to implement at quarantine stations (Moore and Jaykus, 2017). It has been utilized in the diagnosis of bovine Coronavirus, Bovine Viral Diarrhea Virus (BVDV) (Aebischer et al., 2014), human Norovirus (Moore and Jaykus, 2017), and murine Norovirus (Ma L. et al., 2018). The combination of RPA with a lateral flow strip (LFS-RPA) has also been developed for a visible and equipment-free assay for detection of Canine Parvovirus type 2. The amplification is first-performed for 15 min followed by visualization of results with naked eye on the LFS within 5 min. The assay could detect CPV-2a, CPV-2b, and CPV-2c with a detection limit of 1.0 × 10^2^ copies per reaction, which was the same as that of a real-time PCR (Liu et al., 2018). Recently, a cost effective paper-based cell-free transcription–translation reactions for Norovirus has been devised. In this method, a Norovirus sample is first enriched using magnetic bead coated synbodies and viral RNA amplified isothermally using NASBA or RT-RPA. The amplified nucleic acids are then added to paper-based cell-free reactions where Norovirus RNAs are detected by sequence-specific toehold switches. The toehold switches generate the lacZɑ peptide, which produces a purple-colored product after complementation with lacZω. Samples positive for Norovirus can be identified by their purple color following the assay (Ma D. et al., 2018). Overall, the technique has all the features required to become established as a gold standard for enteric virus detection.

#### Polymerase Spiral Reaction (PSR)

PSR technique has been recently developed by Liu et al. (2015) and initially used in the amplification of bla_NIM–1_ plasmid in *E. coli* BL-21 cells. It is more like a conventional isothermal PCR, and is generally carried out at constant temperature of 61–65°C, with modifications in primer design. An exogenous region of 22–25 bases is present at the 5′ end of primers in both the forward and reverse directions. The exogenous region in one primer is in the reverse direction to the exogenous region of the other. The product of this reaction is a complex spiral structure and amplification can be checked by monitoring turbidity and/or fluorescent dye (SYBR Green) based visualization in real-time. The technique has a fine sensitivity of 6 CFU per reaction. Canine Parvovirus 2 detection has been optimized based on the principles of PSR to diagnose the infection caused by various CPV-2 antigenic variants (Gupta et al., 2017). Also, a polymerase cross-linking spiral reaction (PCLSR) that utilized an additional cross-linked primer, has been developed for detection of African Swine Fever virus (ASFV) in pigs and wild boars with a detection limit of 7.2 × 10^2^ copies per μL (Woźniakowski et al., 2017). Recently, a visual assay for accurate detection of PEDV that targeted a conserved region in PEDV ORF3 has been developed and it showed 10- fold higher sensitivity compared to RT-PCR (Wang et al., 2019). The PSR technique provides a convenient and cost-effective alternative for clinical screening, on-site diagnosis and primary quarantine purposes.

#### TaqMan-Probe Based Insulated Isothermal PCR

This is a Rayleigh-Bénard convective PCR based technique wherein denaturation, annealing and extension takes place in different zones of a cylindrical vessel with a constant temperature at the bottom. To reduce the heat loss, the vessel is covered with jacket insulators. DNA denaturation takes place at the bottom, primer annealing in the upper zone and extension in the middle of the vessel. This is easy, cost effective, rapid and sensitive enough to be used as a reliable point-of-care diagnosis technique. With the addition of primers, fluorescently tagged probes are also added for the real-time instant observation of the amplification. This method has been deployed for detection of CPV-2 (Wilkes et al., 2015).

#### Radioactive and Non-radioactive Probes

Use of radioactive and non-radioactive probes for the detection of enteric viral infection is also a promising approach and has been used in many research studies. In this strand of research, Mezger et al. (2014) used a padlock probe and rolling circle amplification (RCA) for rapid detection of Rotavirus. A padlock probe is a simple linear oligonucleotide probe consisting of an end sequence that is complementary to the target sequence and on recognizing the target sequence becomes circularized. These complementary ends are connected through a backbone that has a restriction site with a tagged sequence for detection of the target sequence. When the padlock probe ends the ligation, this complete circle is amplified through RCA. The conventional PCR technique could not amplify highly diverse samples, but padlock probes can be easily multiplexed by increasing the number of padlock probes with the need of only a single recognition site. Designing matching probes and adding it to a padlock probe, can also provide a solution for the detection of emerging viruses, omitting the need for optimization of the process.

## Next Generation Tools for the Detection of Enteric Viruses

### Next-Generation DNA Sequencing (NGS)

Next-generation DNA sequencing is now being increasingly applied in understanding the molecular epidemiology, transmission and characterization of pathogens. Instead of gene-by-gene analysis, large deposits of genes available in the clinical sample can be detected in a single test. Applications of NGS are considered as more resourceful. Thus, it is widely accepted as a diagnostic tool and speedily is being replaced with most other molecular diagnostic technologies and has brought revolution in the diagnosis of pathogens. Various modifications and improvements have brought a huge change in the sequencing and identification of genomes. NGS all started with pyrosequencing on the 454 platform. Improvements and modifications helped in the progression to Illumina, Ion-torrent technology with their improved versions. Now the latest developments have come in the form of nanopore technology. Every improvement has increased the efficiency, reduced the error rate and increased the read sizes, with high levels of data generation. In some typical cases only, this technique has been successful in sorting the problem of genotyping. Next generation shotgun sequencing of swine fecal samples from Germany and other European countries were undertaken to identify the epidemiological data, and also to investigate the exact reason for the outbreak of porcine epidemic diarrhea in these places during 2014. It was thought that similar virus was responsible for outbreak in the United States during 2013. Analysis showed that the virus was recently re-introduced to Germany and Central Europe during 2014. Thus, NGS can also be employed to understand the genetic relationship among isolates recovered during an outbreak (Hanke et al., 2017). Using nanopore sequencing systems, sequencing can be performed on the portable MinION device, the benchtop GridION and the high-throughput, high-sample number PromethION. Recently, nanopore sequencing has proved a revolutionary diagnostic tool in detecting the porcine viral enteric disease complexes (Theuns et al., 2018).

Vector enabled metagenomics has been found to be more beneficial for the identification of viruses, especially in the environmental samples (Rosario et al., 2009, 2012). So, it is quite possible that, with modifications, this technique could be used to detect enteric viruses. Although it is a very good method, its current usage is limited due to high cost. Considering its reliability and performance, modulating it into a cost effective technique should now be given a major focus.

### Mass Spectrometry

The implementation of spectrometry into disease diagnosis has resulted in a significant success. Modern mass spectroscopy techniques were initially developed through efforts of Dempster (1918) and Aston (1919). Later, these were successfully applied to viral diagnosis at the genomic and proteomic levels. In this context, the pathogen could be detected by the development of mass spectrometry–based identification, either via matrix-assisted laser desorption ionization time-of-flight mass spectrometry–based systems (MALDI-TOF MS) or through PCR-electrospray ionization. In the latter approach, ions are generated in the samples, separated by their mass to charge ratio and identified on a detector. The result is then analyzed and compared with a reference database. In the former approach, the pathogen is isolated, whereas in the latter, PCR-electrospray ionization mass spectrometry identifies the nucleic acid composition of multiple PCR-amplified, broadly conserved regions of viral genomes.

The combination of PCR with Mass assay is better than standard methods where there are co-infection with multiple viruses in a single specimen. A novel PCR-Mass assay, combining multiplex PCR with MALDI-TOF MS has been developed and used for the simultaneous detection of eight distinct human enteric viruses (Piao et al., 2012). Another technique exploited for the rapid and specific detection of enteric viruses is Surface Enhanced Raman Spectroscopy (SERS), a Raman Spectroscopic (RS) technique that provides greatly enhanced Raman signals from Raman-active analyte molecules that have been adsorbed onto certain specially prepared metal surfaces. Using SERS, Driskell et al. (2010) detected Rotavirus strains by identifying their molecular signature. Chemometric methods of data analysis, i.e., PLS-DA, facilitated viral classification based on spectral differences. Its direct application in measuring the intricacies and physical properties of the molecules is more advantageous than the indirect usage of reporters tagged with either fluorochrome or radioactive substances. Its major drawback, however, is that it is labor intensive, time consuming, has dependency on operator skills, and thus there is a need for automation.

### Biosensors

**FIGURE 4:**
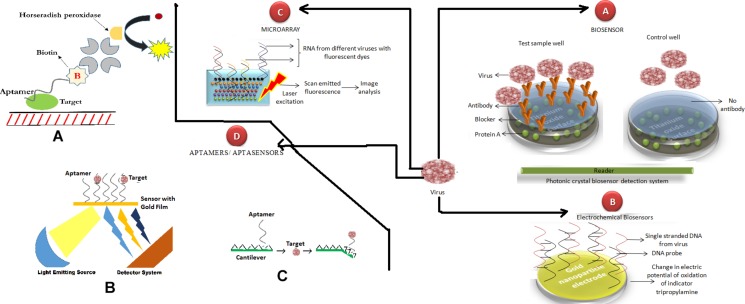
Next generation techniques used in the detection of enteric viruses. Recent techniques like (A) Biosensors, (B) Electrochemical sensor, (C) Microarray, (D) Aptamers/Aptasensors etc. have been employed for the detection of enteric viruses. Further, in aptamer based diagnostics: **(A)** Aptamer-linked immunosorbent assay, ALISA (Alike ELISA except use of antibodies), **(B)** SPR-aptasensors, and **(C)** cantilever aptasensors are in use for detecting various viruses including norovirus strains.

Biosensors provide rapid means for the detection of viruses in a variety of samples. The fundamental aspect in the construction of biosensors is the selection of the right kind of membrane. The organic and inorganic materials have been used by many researchers, e.g., sodium azide, nitrocellulose, polyether-sulfonate and nylon. The main objective in all the biosensors is the detection limit, although the mode of detection may be the whole virus or any protein (Figure 4). Pineda et al. (2009) fabricated a biosensor for the rapid detection of porcine Rotavirus. The confirmation of the attachment of Rotavirus onto the developed biosensor was done by using scanning electron microscopy, which revealed the typical characteristics of Rotavirus particles observed in anti-rotavirus IgG coated wells. A few biosensors include a Bovine Viral Diarrhea Virus sensor, developed using nitrocellulose membranes linked antibodies with a detection limit of 10^3^ CCID/ml (Luo et al., 2010), and a feline Calicivirus biosensors developed using a polyacrylamide membrane, with a detection limit of 1.6 × 10^5^ PFU/ml (Liu et al., 2007). Pineda et al. (2009) rapidly detected the specific porcine Rotavirus using photonic crystal biosensors in water resources. Connelly et al. (2012) developed a micro-total analysis system for virus detection as an integrated microfluidic device. Feline Calicivirus was used as a model for human Norovirus, as it is from same family but non-pathogenic to humans. The device combines sample pre-concentration and liposome-based signal amplification for the detection of enteric viruses present in environmental water samples. This integrated approach decreases time demands associated with conventional cell culture and reduces contamination risk associated with PCR-based techniques. Antibodies raised in rabbits were tagged to liposome, and pre-concentration of the virus liposome was achieved followed by liposome lysis and release of a fluorescent dye which is then quantified. The detection limit of the microfluidic system was found to be 1.6 × 10^5^plaque forming unit per mL (Connelly et al., 2012). In another study, Zhang et al. (2015) developed a precise and rapid method for the detection of Rotavirus and Hepatitis A virus using F_0_F_1_-ATPase molecular motor biosensor. A molecular motor was constructed by connecting the probe to F_0_F_1_-ATPase with the help of a biotin-streptavidin system. The probe was designed by using the Rotavirus VP7 gene and the chromatophore used was extracted from thermophilic bacteria (*Thermomicrobium roseum*). The sensitivity of this system was 0.005 and 0.01 ng/ml for RV and HAV, respectively, and showed no cross reactivity. Moreover, this system takes less than 1 h to specifically detect the food-borne enteric virus (Zhang et al., 2015).

In a *photoluminescence-based immuno-biosensor*, a Graphene oxide array was developed to detect the enteric pathogens, and Rotavirus was specifically identified by employing the Fluorescence Resonance Energy Transfer (FRET) (Jung et al., 2010).

The portability and simplicity of electrochemical DNA sensor makes *Electrochemical biosensors* a potential candidate for a point-of-care development (Figure 4; Zhang et al., 2016; Chen et al., 2017). These are more sensitive and depend on the hybridization of DNA probes immobilized on the gold nanoparticles to the specific complementary single stranded DNA target sequences. The result is detected through a change in the electric potential of oxidation of the indicator, tripropylamine. Its detection limit has been found to be low at 6.94 fg/uL. The major advantage of this technique is that even a low concentration of ions and molecules does not affect its efficiency. On the other hand, this is an expensive technique, so it is unlikely to be practical for clinical use in poor countries. In addition, its reference database needs to be regularly updated and it is not sensitive enough to detect every mutation. Its efficiency has been checked in the detection of Hepatitis A virus cDNA (Manzano et al., 2018). Recently, a highly sensitive electrochemical biosensor has been devised by coating a high affinity binding peptide of Norovirus over gold electrode (Baek et al., 2019). It provided a detection limit of 1.7 copies/mL, which was three-fold lower than the previous reported methods. For easy detection with high sensitivity, electrochemical biosensors would be more helpful for the on-field detection of enteric viruses. Its ease-of-use makes it particularly suitable for mass utilizations in case of endemic outbreaks.

### Microarrays

Microarrays are highly suitable for bulk analysis, surveillance and vigilance, and have also been used for tracking the reservoirs in endemic regions. They were used by Wang et al. (2002) for the simultaneous detection of hundreds of viruses. A similar study was carried out by Buss et al. (2015) using a FilmArray GI Panel pouch that contained an internal nucleic acid extraction control and a PCR control. The FilmArray GI Panel test consists of automated nucleic acid extraction, reverse transcription, amplification and analysis, with results available in 1 h per run per specimen. A DNA microarray was developed by Martínez et al. (2015) and 14 reference gastrointestinal viruses were tested for the validation of the developed DNA microarray. Five different viral species were identified using the microarray when analyzing clinical samples: HAdV-F, HAdV-A, HPeV, HBoV, and several TTVs (Figure 4).

In one European study, multiple organisms were detected in 30% of positive samples by the FilmArray GI Panel, where routine methods were able to identify only 1% of samples (Buss et al., 2015). Also at Mayo, EPEC topped the list, with EAEC the fourth most commonly diagnosed potential agent (EPEC, toxigenic *C. difficile*, Sapovirus, EAEC, Norovirus, Campylobacter, and ETEC). The high rate of EPEC and EAEC detection in the stools of patients but also in controls has previously been reported by others (Jensen et al., 2014; Gomes et al., 2016). Recently, genotyping of Norovirus and Hepatitis A viruses was conducted using DNA microarray platform and it could detect less than 10 cRNA transcripts (Quiñones et al., 2017).

The FilmArray GI Panel and molecular diagnostics in general have another inherent limitation: viral, bacterial and parasite nucleic acid may persist *in vivo* independently of organism viability (Spina et al., 2015). Discrepancies between the FilmArray GI Panel and other microbial identification methods may also be due to the inability to differentiate species reliably with standard phenotypic microbial identification methods.

### Aptamers

As an alternate to antibodies, aptamers (a stretch of oligonucleotides or peptide) received a considerable attention of researchers globally. Carrying all the uniqueness of antibodies, they are thermostable and economical, thus making them more suitable to be used in the resource limited settings (Song et al., 2012). For effective diagnosis, aptamers are coupled with RT-PCR, alike ELISA they are used as Aptamer linked immune sorbent assay, SPR based and cantilever based aptasensors (Figure 4). The high sensitivity of aptasensors is advantageous and thus has potential for its use in point-of-care detection system, since they are portable, easy, and offer quick detection (Kieboom et al., 2015). This method has been well utilized in the diagnosis of Norovirus strains effectively (Escudero-Abarca et al., 2014). Bovine Viral Diarrhea Virus has also been identified using aptamers with a detection limit of 800 copies/mL that is comparable to real-time PCR (Park et al., 2014). An aptamer based on *in situ* capture RT-qPCR assay for detection of human noroviruses from clinical samples has recently been devised (Liu D. et al., 2019).

## Prospective Techniques

There are many elegant, sensitive and specific techniques that have been used in the diagnosis of viral pathogens, but their applications have not been explored in the detection of enteric viruses. These techniques can be further modified to normalize the suitable enteric virus identification and can also be brought into use for efficient diagnosis. Some of these are given below:

Tissue-based immunoassay and slide ELISA (sELISA) are ELISA-based solid phase assays. The former is performed on cellulose membrane and the latter on slides, where heat fixed viral particles are diagnosed. Various plant viruses have been diagnosed with tissue immunoblotting assay (TIBA) (Hancevic et al., 2012) and Newcastle Disease Virus through sELISA (Desingu et al., 2014). These could prove to be very simple methods for the detection of enteric viruses. Similarly, proximity extension assay (Assarsson et al., 2014) is very sensitive for the identification of pathogens. This uses specifically designed primers to obtain a complex structure or pattern which could be visualized using agarose gel or via real-time monitoring using fluorescence dye. Besides, proximity ligation assay is an immobilized DNA aptamer molecule-based amplification of the connector of two closely placed DNA molecules followed by ELISA, which increases the sensitivity many fold. These assays are especially useful for the detection of pathogens which are present in limited quantities in clinical samples (Lundberg et al., 2011).

Peptide nucleic acids (PNAs) can also be another potential diagnostic for the enteric viruses. PNAs are synthetic oligonucleotides in which the native sugar-phosphate backbone of DNA is replaced with amino acids, especially N-(2-aminoethyl)-glycine units are linked by amide bonds, and to which purine and pyrimidine bases are attached, giving it the properties both of nucleic acids as well of amino acids (Siddiquee et al., 2015). PNAs are sensitive to detect mutants even in low amounts and performance is next to next-generation deep sequencing (Cai et al., 2016; Wang et al., 2017). PNA-PCR assay is performed to estimate the sensitivity of PNAs on plasmid mixtures comprised of varying amounts of mutant and wild-type plasmids (Hong et al., 2016). PNAs have many applications, including PNA-based probes for fluorescence *in situ* hybridization, PNA-based transcriptomic studies and single-nucleotide polymorphism (SNP) arrays, and therapeutic inhibition of mRNA/micro-RNAs.

Another potent application could be found in NASBA-CRISPR cleavage that exploits the sequence-specific nuclease activity of CRISPR/CAS9 in the detection and discrimination of viral strains. NASBA-CC influences Cas9 to cleave DNA in the vicinity of NGG protospacer adjacent motif (PAM). Using this property, RNA is amplified in the presence of a reverse primer and full-length RNA product generation activates the sensor H whereas truncated RNA does not. Pardee et al. (2016) established this new technique to identify the Zika virus, by coupling the NASBA amplification and CRISPR/CAS9 property. Certain strains are more prevalent in specific regions, like the Brazilian strain of the Zika virus. A high number of cases were reported of fetal microcephaly and Guillain-Barré syndrome due to mutations (Calvet et al., 2016; Mlakar et al., 2016). This strategy is very useful for rapid identification of new strains with single mutations at low cost.

Another new approach could be a droplet digital PCR (ddPCR) assay for diagnosis of enteric viruses. This approach has shown promising results for sensitive detection of PCV3 cap gene. The assay showed a detection limit of 1.68 ± 0.29 copies of PCV3 DNA per reaction, which was approximately 10-fold sensitive compared to RT-qPCR (Zhang et al., 2019).

## Conclusion

Successful detection of enteric viruses or other pathogens depends on the factor whether they can detect cultivable and fastidious pathogen entities. Despite the great success in the field of pathogen diagnosis tools, approaches that are widely applicable, inexpensive and simple are missing. Moreover, mixed infections are also a hurdle in disease diagnosis. Shortcomings need to be eliminated in all the methods, including cell culture, molecular based, immunological and other advanced methods. Multiplex systems offer rapid detection and future perspectives will be the multi-target amplification tests which currently require multiple testing modalities. These will include multiplex and arrayed singleplex systems. The choice of the correct target site is vital for success in molecular detection, but to find such targets is not an easy task. The evaluation of non-cultivable viral strains to study the epidemiological perspectives and disease diagnosis is another future target. Metagenomics has revealed many new viral entities in the recent past and has seemed to be a promising approach; without a low cost rapid detection technology, however, its use cannot be expanded. Biosensor probe-based methods, however, can replace the need for NGS. The use of molecular diagnostics and advanced tools such as biosensors promises new insights into the etiologies of infectious diarrhea.

We are confident that these advanced diagnostic techniques will in time refine our understanding of epidemiology and vaccine development against emerging diarrhea and other enteric diseases. The perspective always is to develop an easy and streamlined process to detect the enteric viruses in diarrheic animals in the farm itself, or to deliver a warning to adopt necessary precautions or prescriptions from veterinarians. One of the methods can include a dry SDS gel-based resolution of viral proteins, just by soaking the setup in water and by applying an electric current, fluorescent electropherotypes patterns can help in the identification of the specific viral infection. We can even consider spiked fecal samples suspension in buffer and their treatment with affinitive molecules, like especially designed monoclonal antibodies attached with fluorescent tags, which would bind to the specific target viruses and on excitation at an appropriate wave-length light will appear as glowing floating points in the dark. Rapid chromatographic methods could be in line for the differentiation of various serotypes for viruses like Rotavirus, Norovirus, Adenovirus, etc. For real-time detection, an innovative idea could be prescribing digestible antibodies tagged with fluorochromes that bind to the enteric viruses, as well as ingested micro-cameras which could detect virus when passing through the gut. This will rather help in understanding the better diagnosis of the enteric viruses.

## Author Contributions

All authors substantially contributed to the conception, design, analysis, and interpretation of the data, checking and approving the final version of the manuscript, and agreed to be accountable for its contents. YM, AKV, DB, NKu, and NT initiated the review compilation. AA-M, ANV, MH, KB, and SG updated the various sections. KD reviewed the Indian Scenario. KK designed the tables and the figures. YM, MH, SG, KB, RS, AA-M, and NKo overviewed, thoroughly checked, and finally edited the whole manuscript.

## Conflict of Interest Statement

The authors declare that the research was conducted in the absence of any commercial or financial relationships that could be construed as a potential conflict of interest.
